# Supplementary material for: A Humanized Yeast Phenomic Model of Deoxycytidine Kinase to Predict Genetic Buffering of Nucleoside Analog Cytotoxicity
Source: Genes (Basel). 2019 Sep 30;10(10):770. doi: 10.3390/genes10100770 (PMC6826991; doi:10.3390/genes10100770)
Supplement: Supplementary file 1 [file genes-10-00770-s001.zip › Additional_Files/Additional_File7_GOTermHeatmaps.zip!/Additional_File7_GOTermHeatmaps/carbon-nitrogen_ligase_activity,_with_glutamine_as_amido-N-donor.pdf]

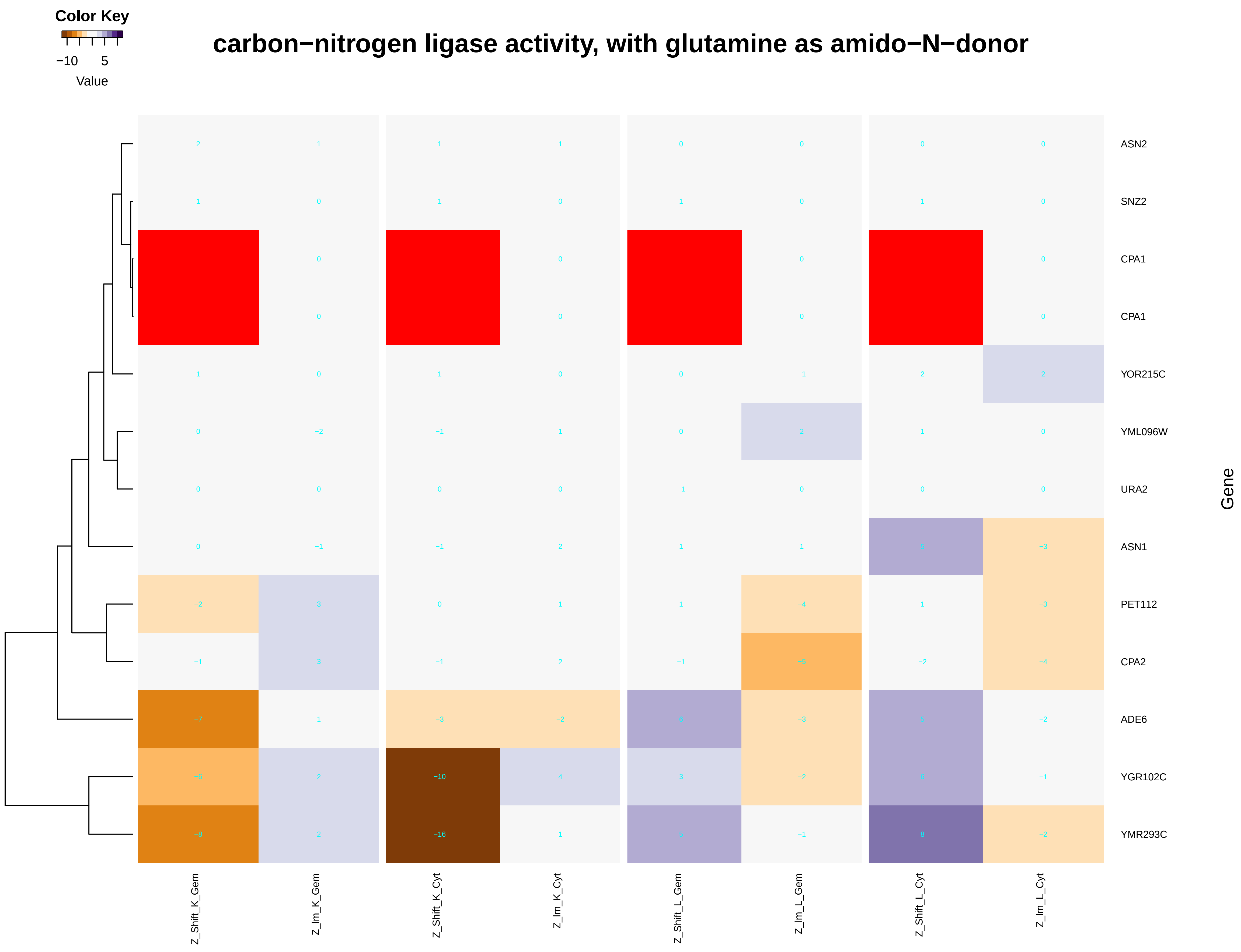

Color Key
−10
5
Value
carbon−nitrogen ligase activity, with glutamine as amido−N−donor
ASN2
SNZ2
CPA1
CPA1
YOR215C
YML096W
URA2
ASN1
PET112
CPA2
ADE6
YGR102C
YMR293C
Gene
Z_Shift_K_Gem
Z_Im_K_Gem
Z_Shift_K_Cyt
Z_Im_K_Cyt
Z_Shift_L_Gem
Z_Im_L_Gem
Z_Shift_L_Cyt
Z_Im_L_Cyt

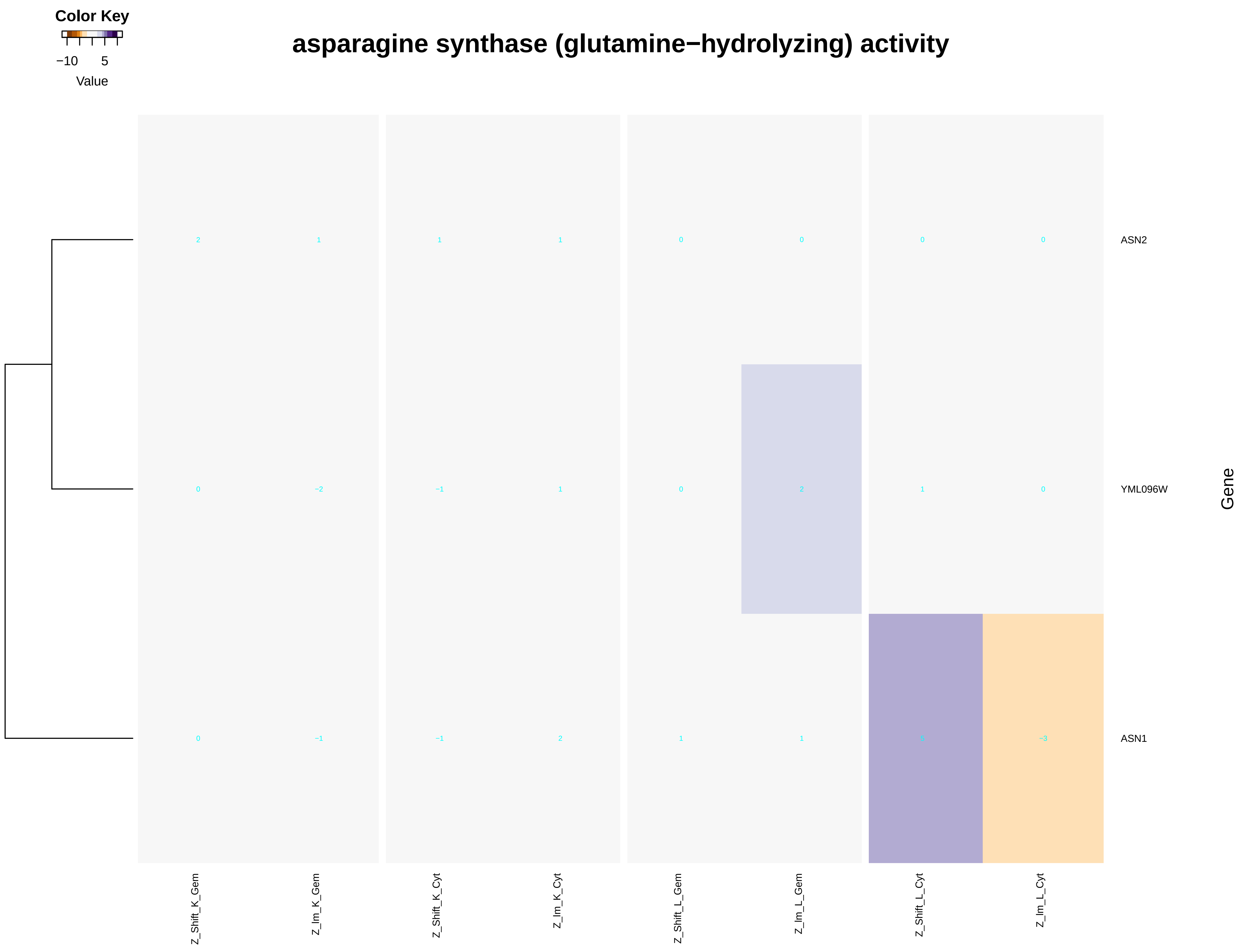

Color Key
−10
5
Value
asparagine synthase (glutamine−hydrolyzing) activity
2
1
1
1
0
0
0
0
ASN2
0
−2
−1
1
0
2
1
0
YML096W
0
−1
−1
2
1
1
−3
ASN1
Gene
Z_Shift_K_Gem
Z_Im_K_Gem
Z_Shift_K_Cyt
Z_Im_K_Cyt
Z_Shift_L_Gem
Z_Im_L_Gem
Z_Shift_L_Cyt
Z_Im_L_Cyt

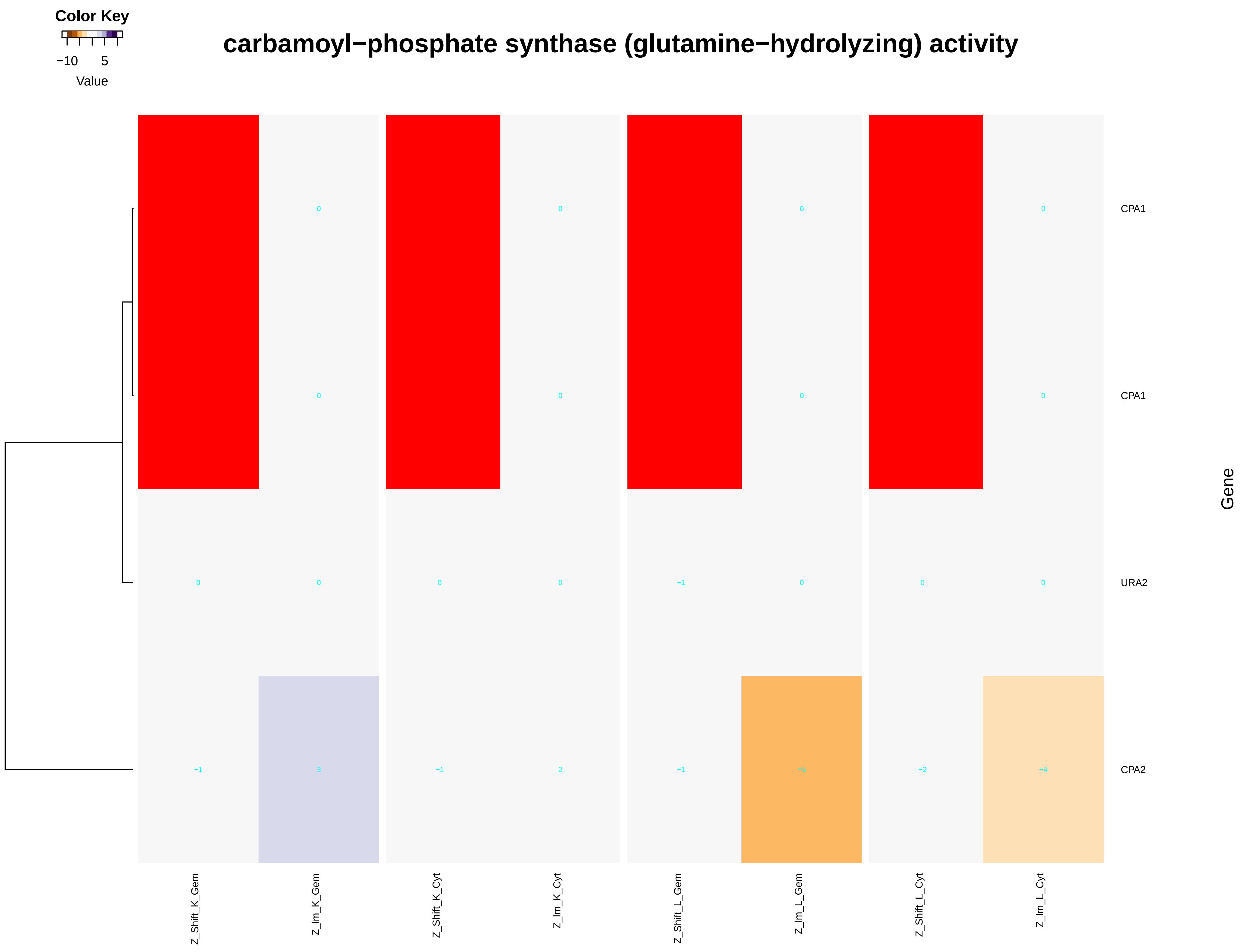
Color Key
−10 5
Value
carbamoyl−phosphate synthase (glutamine−hydrolyzing) activity
CPA1
CPA1
URA2
CPA2
Gene
Z_Shift_K_Gem
Z_Im_K_Gem
Z_Shift_K_Cyt
Z_Im_K_Cyt
Z_Shift_L_Gem
Z_Im_L_Gem
Z_Shift_L_Cyt
Z_Im_L_Cyt

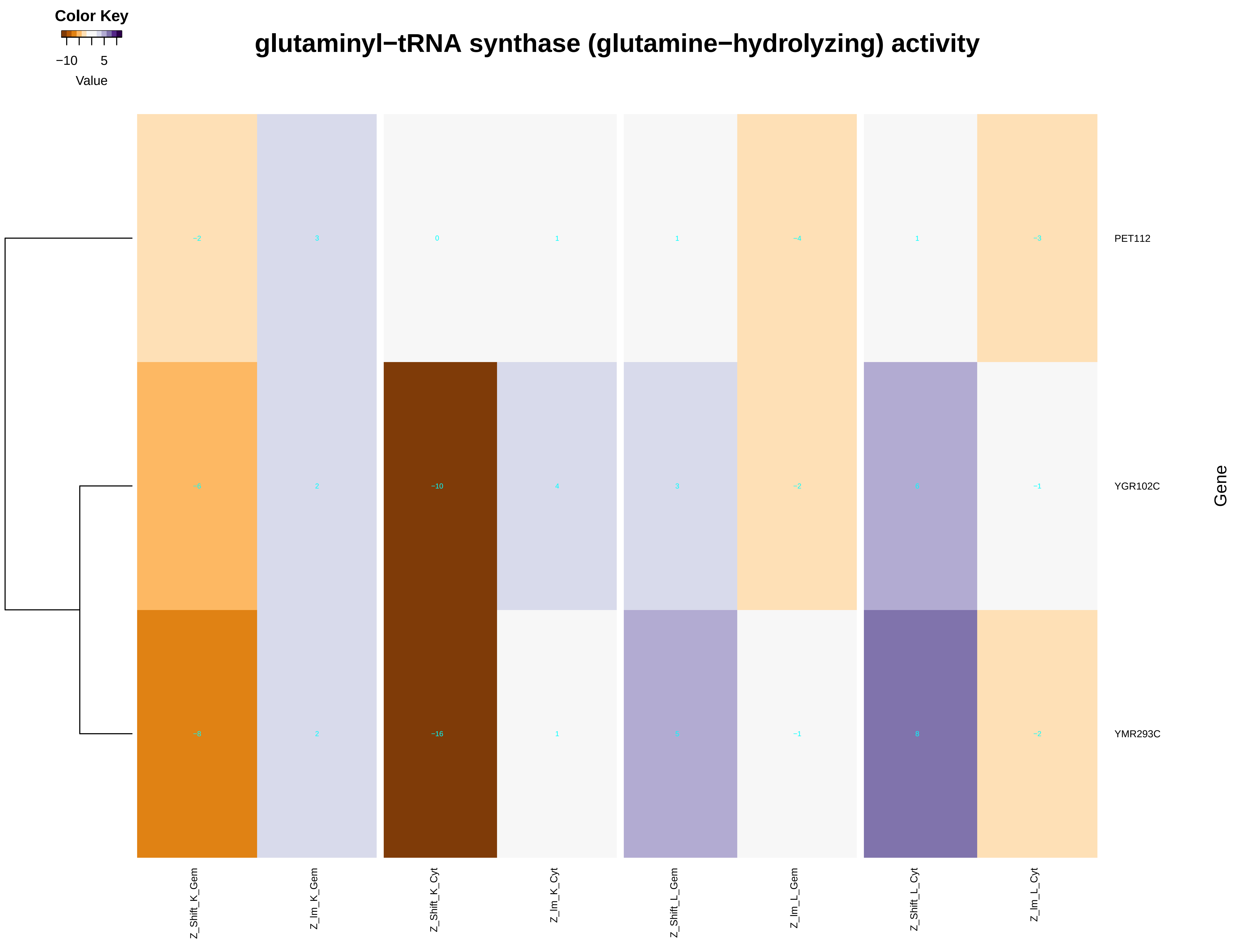
Color Key
−10
5
Value
glutaminyl−tRNA synthase (glutamine−hydrolyzing) activity
PET112
YGR102C
YMR293C
Gene
Z_Shift_K_Gem
Z_Im_K_Gem
Z_Shift_K_Cyt
Z_Im_K_Cyt
Z_Shift_L_Gem
Z_Im_L_Gem
Z_Shift_L_Cyt
Z_Im_L_Cyt
